# Maternal adipokines and insulin as biomarkers of pregnancies complicated by overweight and obesity

**DOI:** 10.1186/s13098-016-0184-y

**Published:** 2016-09-13

**Authors:** Joice Monaliza Vernini, Jusciéle Brogin Moreli, Roberto Antônio Araújo Costa, Carlos Antonio Negrato, Marilza Vieira Cunha Rudge, Iracema Mattos Paranhos Calderon

**Affiliations:** 1 Graduate Program in Gynecology, Obstetrics and Mastology, Botucatu Medical School, São Paulo State University/UNESP, Botucatu, São Paulo Brazil; 2 Department of Gynecology and Obstetrics, Botucatu Medical School, São Paulo State University/UNESP, Botucatu, São Paulo Brazil; 3 Bauru’s Diabetics Association, Internal Medicine, Bauru, São Paulo Brazil; 4 Faculty of Medicine of Botucatu, Universidade Estadual Paulista-UNESP, District Rubião Jr. s / n, Botucatu, São Paulo, CEP 18618-000 Brazil

**Keywords:** Adipokines, Pregnancy, Obesity, Insulin resistance, Hyperglycemia

## Abstract

**Background:**

Maternal obesity is associated with several adverse pregnancy outcomes. This study was conducted aiming to evaluate maternal levels of adipokines and insulin in pregnancies complicated by overweight and obesity and its correlations with maternal and fetal outcomes.

**Methods:**

This cross-sectional study included 72 mother–newborn pairs. Mothers were classified as having normal weight (n = 23), overweight (n = 18), and obesity (n = 31). Maternal adiponectin, leptin, resistin and insulin levels at the end of pregnancy were compared among groups and correlated with maternal and perinatal outcomes. Data were analyzed by ANOVA and correlation tests, with a *p* value <0.05 being considered as significant.

**Results:**

Obese pregnant women showed higher leptin levels (*p* = 0.0021). Leptin levels were positively correlated with prepregnancy body mass index—BMI (r = 0.57), gestational (37 or 38 weeks of gestation) BMI (r = 0.39), hypertension (r = 0.27), and hyperglycemia (r = 0.30), and negatively associated with newborns’ abdominal circumference (r = −0.25). Adiponectin concentrations were negatively correlated with gestational BMI (r = −0.29) and newborns’ cephalic circumference (r = −0.27) and positively correlated with birth weight (r = 0.23). Insulin concentrations correlated positively with prepregnancy BMI (r = 0.38), gestational BMI (r = 0.24) and maternal hyperglycemia (r = 0.26).

**Conclusions:**

Our findings support the relationship between markers of obesity and maternal–fetal outcomes. Maternal insulin and adipokines levels showed an independent relationship with mother and newborns outcomes, respectively. In this studied population, the results indirectly reinforce the importance of maternal weight control before and during pregnancy to avoid adverse outcomes to mother and their newborns.

## Background

In recent years the endocrine nature of adipose tissue has been widely recognized. Its cells synthesize and release a number of substances such as many types of adipokines. Adipokines are primarily secreted from adipose tissue and function as important regulators of appetite, glucose homeostasis, immune function, arterial blood pressure and blood coagulation [1–3].

Growing evidence has pointed to a putative and causative relationship between obesity and inflammation. An increase in adipose tissue and consequently in adipokines production triggers a series of physiopathologic processes associated to the genesis of obesity-related pathologies. The production and release of adipokines or inflammatory cytokines characterize obesity as a chronic inflammatory state [1, 4–7]. In the last decade, different adipokines types have been identified, such as leptin, adiponectin, adipsin, resistin, tumoral necrosis factor alpha (TNF-α), plasminogen activator inhibitor-1 (PAI-1), interleukin (IL) 1β, IL-6 and IL-8, insulin-like growth factor 1 (IGF-1), monocyte chemo attractant protein-1 (MCP-1) and visfatin. Except for adiponectin, the production and secretion of these substances is proportional to the degree of obesity. A number of these adipokines, such as adiponectin, leptin, TNF-α, resistin, PAI-1, IL-6 and MCP-1, were found to be directly related to insulin resistance and associated morbidities [8–11].

In pregnant women, several metabolic changes such as progressive increase and redistribution of maternal adipose tissue occur to ensure continuous nutrient supply to fetal development. This affects a series of metabolic functions that characterize a state of dysmetabolism with consequent increase of the risk for adverse pregnancy outcomes [5, 8, 12, 13]. Hormones produced by the placenta, particularly in the second and third trimesters of pregnancy, generally antagonize insulin action, thereby establishing insulin resistance in parallel with an increase in the production of this hormone [14, 15]. It is thought that adipokines participate in the process of insulin resistance during pregnancy, that may be related to the occurrence of gestational diabetes mellitus (GDM), preeclampsia (PE) and intrauterine growth restriction (IUGR). Insulin resistance in pregnancy has been particularly associated with adiponectin and leptin, which are secreted only by the adipose tissue, and IL-6 and TNF-α, which are also secreted by the adipose tissue and other cells [12, 14, 16].

Among adipokines, leptin is known as the “satiety hormone” given that it inhibits food intake and stimulates energy expenditure through activation of receptors in the hypothalamus. High leptin levels are found in obese people, possibly because they exhibit resistance to the action of this hormone [17, 18]. Adiponectin and resistin are adipocytokines with antagonistic actions. Adiponectin increases insulin sensitivity, whereas resistin increases insulin resistance [18–20].

Obesity and pregnancy play individual roles in the development of chronic inflammation and insulin resistance, and their combination can be particularly harmful to the mother and the fetus [21, 22]. Many gaps exist in the understanding the role of obesity during pregnancy and the causal relationship with associated disorders. It is not known which problems arise from obesity itself and which are a result of unhealthy lifestyle such as sedentarism and use of an inadequate diet, which, in turn, can promote obesity [11, 23, 24]. Likewise, the lack of individualization in the studies, considering only overweight or obese mothers without hypertension, hyperglycemia or insulin resistance conditions, may change these results.

To clarify this scenario, adipokines deserve deeper investigation given their potential role as biomarkers of obesity and insulin resistance in pregnancy, and therefore perinatal outcomes.

The present study evaluated the serum levels of adipokines (adiponectin, leptin and resistin) and insulin in overweight and obese pregnant women and correlated them with maternal and perinatal outcomes.

## Methods

### Study design and subjects

We conducted a cross-sectional study with 72 mother-newborn pairs referred and treated in Diabetes and Pregnancy Care Service of Botucatu Medical School—UNESP (SEDG-FMB/UNESP). The research was approved by the Human Research Ethics Committee of the Medical School of Botucatu (CEP-FMB/UNESP). All included subjects signed the written informed consent.

The women were classified according to prepregnancy BMI into normal weight (18.5–24.9 kg/m^2^; n = 23), overweight (25.0–29.9 kg/m^2^; n = 18) and obese (≥30.0 kg/m^2^; n = 31) [24]. Underweight women and those with multiple pregnancies or malformed fetus were excluded from the study.

### Studied variables

Pregnant women enrolled in the study were examined at their first prenatal (before the 20th gestational week) and pre-birth visit (37–38th gestational weeks). They provided information on their age, number of pregnancies, prepregnancy BMI, smoking habits and preexistence of chronic arterial hypertension and diabetes mellitus. In the pre-birth visit (37–38th gestational weeks) maternal body weight, height and blood pressure were evaluated according to the standardization of the Brazilian Ministry of Health [25]. Gestational BMI, weight gain, hypertension complicating pregnancy (yes/no), GDM and mild gestational hyperglycemia (MGH) (yes/no) [26–28] were associated with maternal adiponectin, leptin, resistin and insulin levels in the pre-birth period (37–38th gestational weeks).

The newborns were examined in order to evaluate gestational age at birth by the New Ballard score, weight to gestational age ratio, ponderal index [(weight/length^3^)*100], length as well as head, thoracic and abdominal circumferences. These evaluations have been conducted by a specialized pediatrician and followed the normalization of our Service, including the New Ballard score. All these variables also were associated with maternal adiponectin, leptin, resistin and insulin levels in the pre-birth period (37–38th gestational weeks).

### Collection of maternal blood and analysis of adipokines

Maternal blood samples were collected at in Vacutainer tubes (Becton–Dickinson, USA) and centrifuged at 4 °C for 15 min at 1000×*g*. The serum samples were stored at −80 °C until adiponectin, leptin, resistin and insulin analysis were performed using multiplex ELISA assays (Millipore Corporation, Massachusetts, USA).

### Subjects’ follow-up

Pregnant women with prepregnancy type 1 and type 2 diabetes or those who showed risk of GDM or MGH development [27, 28] were referred to the SEDG-FMB/UNESP. According to the standard protocol used by our service, those pregnant women that did not have the diagnosis of diabetes, performed a 75 g oral glucose tolerance test and had their glycemic profile (GP) determined, between 24 and 26 gestation weeks, to confirm or not the diagnosis GDM or MGH [27, 28]. Pregnant women with MGH presented positive screening for GDM, negative diagnosis for GDM but with hyperglycemia detected in the GP [27]. According to our center’s routine protocol, diabetic pregnant women (type 1-DM or type 2-DM) were immediately managed with glycemic control, individualized nutritional intervention, and light to moderate-intensity exercise program (most frequently walking for 30 min five times a week). Insulin therapy was always maintained (DM-1) or introduced in substitution to oral antidiabetics (DM-2) [27]. Pregnant women who were normoglycemic, but were overweight or obese, received counseling about the importance of lifestyle changes to prevent GDM and MGH, and were promptly assigned to individualized nutritional guidance, walking for 30 min five times a week for weight control during pregnancy. Regardless of these preventive measures, all normoglycemic pregnant women underwent glucose tolerance (75 g-OGTT) and glycemic profile (GP) testing between 24 and 28 weeks of pregnancy for confirmation or ruling out of GDM and MGH. Pregnant women with confirmed GDM or MGH were treated according to the same protocol, relative to diet and exercise, aiming to achieve glycemic control, and insulin therapy was introduced when necessary. Glycemic control and management of diabetes were evaluated by 24-h GP (fasting, pre- and post- prandial glycemic levels) performed at 2-week intervals until week 32, and weekly until delivery. The glycemic mean (GM), calculated by the average of the glycemic levels evaluated in the GP, was used to classify the quality of maternal glycemic control in adequate (GM < 120 mg/dL) or inadequate (GM ≥ 120 mg/dL) [27–30].

### Statistical analysis

Data obtained in the medical visits were regularly exported to Microsoft Excel spreadsheets. Statistical analyses were performed using the SAS package for Windows, version 9.2. Adipokines and insulin data were evaluated by ANOVA, followed by the Tukey–Kramer test for multiple comparisons. Data correlation was tested using Pearson or Spearman correlation tests according to distribution normality. Finally, a multiple regression analysis was performed to identify the independent factors to mother and newborn outcomes. The statistical significance was set at 0.05 for all the tests.

## Results

According to prepregnancy BMI, out of the 72 pregnant women evaluated, 23 (31.9 %) had normal weight, 18 (25.0 %) were overweight, and 31 (43.1 %) were obese. Independently of nutritional status, most of the pregnant women had between 20 and 35 years old, and at least two previous pregnancies. All types of arterial hypertension complicating pregnancy were observed in 33 (45.8 %) of studied population. A proportion of 16.7 % was smoker and 48 (66.7 %) exhibited some form of hyperglycemia, which was diagnosed as preexisting diabetes (DM2 = 16.7 %), GDM (50.0 %) or MGH (33.3 %). Hypertension and diabetes were more frequently associated with overweight and obesity (Table 1).

**Table 1:** Profile of the pregnant women, according to prepregnancy BMI classes

	Normal weight (N = 23)	Overweight (N = 18)	Obese (N = 31)
Age (year)			
<19	9 (39.1)	1 (5.5)	0 (0.0)
20–35	12 (52.2)	12 (66.7)	24 (77.4)
>35	2 (8.7)	5 (27.8)	7 (22.6)
Number of pregnancies			
1	9 (39.1)	3 (16.7)	3 (9.7)
≥2	14 (60.9)	15 (83.3)	28 (90.3)
Smoking	5 (21.7)	0 (0.0)	7 (22.6)
Hypertension (n = 33)	3 (13.0)	9 (50.0)	21 (67.7)
Chronic hypertension	0 (0.0)	1 (3.0)	10 (30.3)
Preeclampsia	2 (6.0)	6 (18.2)	8 (24.2)
Chronic hypertension + PE	0 (0.0)	0 (0.0)	6 (18.2)
Gestational hypertension	1 (3.03)	2 (6.1)	3 (9.7)
Diabetes mellitus (n = 48)	6 (26.1)	11 (61.1)	31 (100.0)
DM**-**2	0 (0.0)	3 (27.3)	5 (16.1)
GDM	2 (33.3)	4 (36.4)	18 (58.1)
MGH	4 (66.7)	4 (36.4)	8 (25.8)

*BMI* body mass index, *PE* preeclampsia, *DM-2* type 2 diabetes mellitus, *GDM* gestational diabetes mellitus, *MGD* mild gestational hyperglycemia

**Fig. 1:**
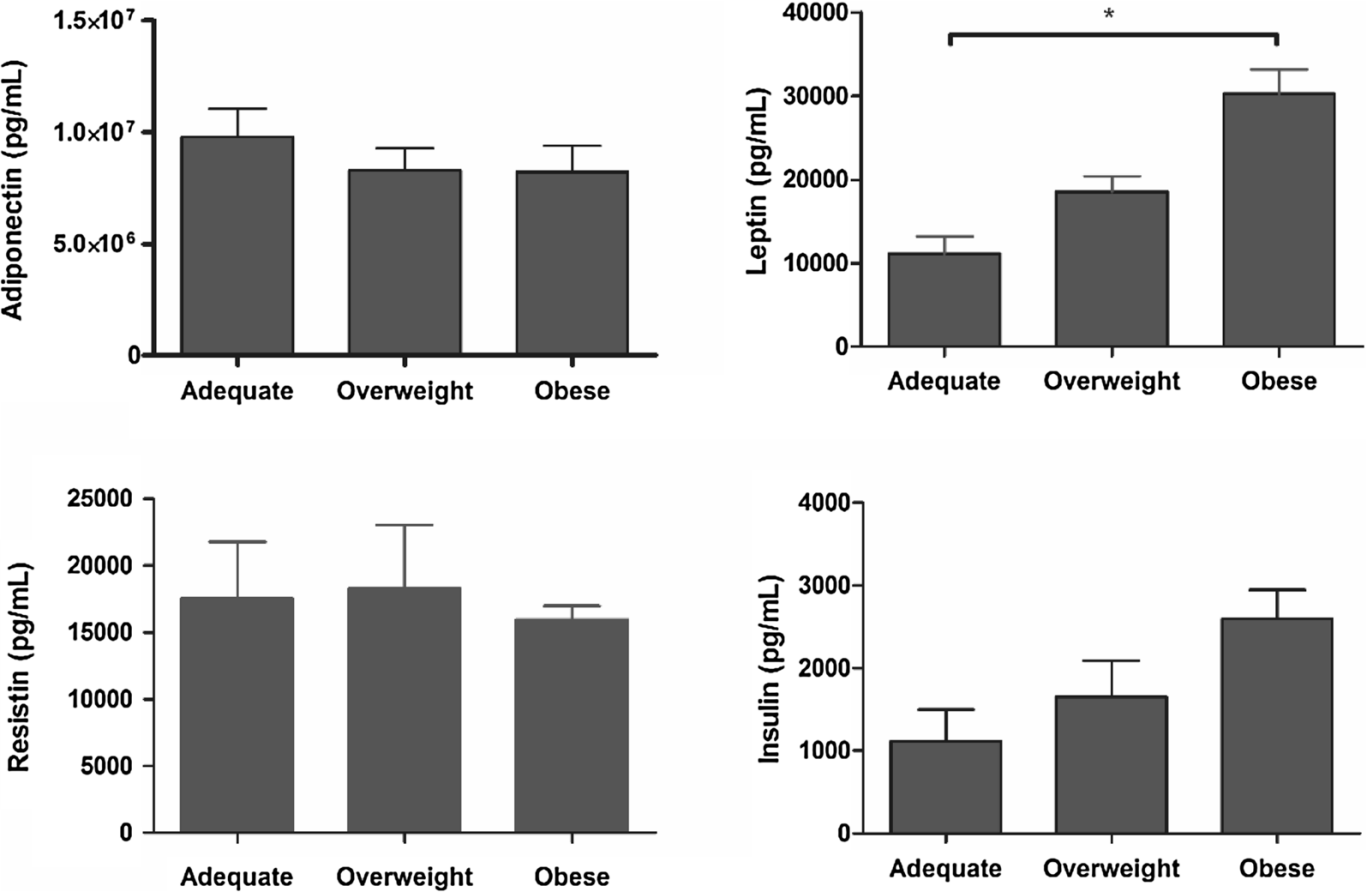
Adiponectin, leptin, resistin and insulin levels according to weight classes of the pregnant women. (Values as mean ± standard error of the mean; *p < 0.05—ANOVA, followed by the Tukey–Kramer test)

Maternal and newborn outcomes are shown in Table 2. As indicated by gestational BMI, out of the 72 pregnant women evaluated at the end of pregnancy, 17 (23.6 %) of the women had normal weight, 17 (23.6 %) were overweight, and 38 (52.8 %) obese. Considering gestational BMI, 8/23 (34.8 %) prepregnancy normal weight were classified as overweight or obese, 16/18 (88.9 %) prepregnancy overweight as overweight or obese, and 31/31 (100.0 %) prepregnancy obese were overweight or obese. Of the 72 newborns, 97.2 % were born to term, and 80.6 % had normal weight. Only one was underweight (<2500 g) and 8.3 % were classified as large for gestational age. Figure 1 shows adiponectin, leptin, resistin, and insulin levels according to weight status. Compared to normal weight pregnant women, leptin levels were significantly higher in obese women (*p* = 0.0021).

**Table 2:** Maternal and newborn outcomes, according to prepregnancy BMI classes

	Normal weight (N = 23)	Overweight (N = 18)	Obese (N = 31)
Maternal			
Gestational BMI (kg/m^2^)			
Adequate	15 (65.2)	2 (11.1)	0
Overweight	6 (26.1)	9 (50.0)	2 (6.5)
Obesity	2 (8.7)	7 (38.9)	29 (93.5)
Newborn			
GA at birth (new Ballard score)			
<37 weeks	0	1 (5.6)	1 (3.2)
≥37 weeks	23 (100.0)	17 (94.4)	30 (96.8)
Weight (g)			
<2500	0	0	1 (3.2)
2500–4000	21 (91,3)	17 (94.4)	27 (87.1)
≥4000	2 (8.7)	1 (5.6)	3 (9.7)
Weight to GA ratio			
Small (SGA)	2 (8.7)	4 (22.2)	2 (6.4)
Adequate (AGA)	19 (82.6)	13 (72.2)	26 (83.9)
Large (LGA)	2 (8.7)	1 (5.6)	3 (9.7)

	Normal weight	Overweight	Obese
	Mean ± SD	Mean ± SD	Mean ± SD
Maternal			
Weight gain (kg)	14.6 ± 4.5	13.8 ± 6.1	10.4 ± 8,1
Newborn			
Weight (g)	3340 ± 462.8	3220.6 ± 430.7	3333.9 ± 492.2
Ponderal index (g/cm^3^)	2.8 ± 0.3	2.8 ± 0.3	2.9 ± 0.3
Length (cm)	48.9 ± 2.5	48.4 ± 1.7	48.5 ± 2,1
Head circumference (cm)	34.5 ± 1.7	34.5 ± 1.5	34.9 ± 1,5
Thoracic circumference (cm)	33.6 ± 1.9	33.0 ± 1.6	33.3 ± 2.2
Abdominal circumference (cm)	31.9 ± 2.0	31.4 ± 1.8	34.9 ± 1.5

*BMI* body mass index, *GA* gestational age

The correlation between maternal outcomes and markers of insulin resistance is shown in Table 3. Adiponectin levels were negatively correlated with gestational BMI (r = −0.29, *p* = 0.013), and leptin levels positively correlated with prepregnancy BMI (r = 0.57, *p* < 0.0001), gestational BMI (r = 0.39, *p* = 0.0007), hyperglycemia (r = 0.31, *p* = 0.0086) and hypertension (r = 0.27, *p* = 0.0196). Insulin levels were positively correlated with prepregnancy BMI (r = 0.38, *p* = 0.0009), gestational BMI (r = 0.24, *p* = 0.0466) and hyperglycemia (r = 0.26, *p* = 0.0252). Resistin levels were not correlated with the evaluated maternal outcomes. Multiple regression analysis confirmed only one independent relationship that is maternal insulin levels with prepregnancy BMI (*p* = 0.0343) and weight gain (*p* = 0.0417), and a tendency with gestational BMI (*p* = 0.0570) (Table 4).

**Table 3:** Correlation analysis, coefficient (r) and p value, for the relationship between adipokine/insulin levels and maternal–newborn variables

		Adiponectin	Leptin	Resistin	Insulin
Maternal					
Prepregnancy BMI^a^	(r)	0.0522	0.5689	−0.1378	0.3843
	*p*	0.6630	*<0.0001*	0.2483	*0.0009*
Gestational BMI^b^	(r)	−0.2893	0.3886	−0.0720	0.2354
	*p*	*0.0137*	*0.0007*	0.5477	*0.0466*
Weight gain^a^	(r)	−0.1742	0.0948	−0.0620	0.0608
	*p*	0.1434	0.4284	0.6047	0.6118
Hypertension^b^	(r)	−0.0669	0.2746	0.0732	−0.0527
	*p*	0.5767	*0.0196*	0.5413	0.6601
DM and MGH^b^	(r)	−0.1389	0.3076	−0.0206	0.2637
	*p*	0.2445	*0.0086*	0.8639	*0.0252*
Newborn					
Weight^a^	(r)	0.2340	−0.1216	−0.1536	0.1097
	*p*	*0.0478*	0.3089	0.1975	0.3590
Length^a^	(r)	−0.1604	−0.2037	−0.1003	0.0750
	*p*	0.1785	0.0862	0.4019	0.5309
Head circumference^a^	(r)	−0.2704	−0.1373	−0.2194	0.0440
	*p*	*0.0216*	0.2501	0.0641	0.7133
Thoracic circumference^a^	(r)	−0.1777	−0.1845	−0.0572	−0.0254
	*p*	0.1354	0.1208	0.6332	0.8325
Abdominal circumference^a^	(r)	−0.1637	−0.2488	−0.0809	0.0049
	*p*	0.1694	*0.0350*	0.4991	0.9677
Ponderal index^a^	(r)	−0.1605	0.0856	−0.1098	0.0655
	*p*	0.1779	0.4744	0.3584	0.5845

Statistically significant results (*p* < 0.05) highlighted in italic

*DM* prepregnancy and gestational diabetes, *MGH* mild gestational hyperglycemia

^a^Pearson’s correlation test

^b^Spearman’s correlation test

**Table 4:** Multiple regression analysis, *p* value, for the relationship between adipokine/insulin levels and maternal-newborn variables

	Adiponectin *p* value	Leptin *p* value	Resistin *p* value	Insulin *p* value
Maternal—intercept	0.0348	0.0109	0.0130	0.0452
Prepregnancy BMI	0.1598	0.9971	0.3654	*0.0343*
Gestational BMI	0.1904	0.7297	0.4166	*0.0570*
Weight gain	0.1181	0.9258	0.3662	*0.0417*
GM at third trimester	0.4076	0.4172	0.8201	0.0894
Newborn—intercept	0.0781	0.0262	0.1369	0.3185
Weight at birth	0.4478	0.2378	0.5439	0.1291
Head circumference	0.1904	0.4507	0.1910	0.9480
Abdominal circumference	0.6045	*0.0270*	0.4548	0.1522
Ponderal index	0.6533	0.1536	0.7648	0.8645

Statistically significant results (*p* < 0.05) highlighted in italics

*BMI* body mass index, *GM* glycemic mean

Relative to newborn outcomes, maternal adiponectin levels were positively correlated with newborns weight (r = 0.23, *p* = 0.0487) and inversely correlated with newborns head circumference (r = −0.27, *p* = 0.0216). Leptin levels were inversely correlated with abdominal circumference (r = −0.25, *p* = 0.0350). Maternal resistin and insulin levels were not correlated with newborns measures (Table 3). In the multiple regression analysis, only leptin levels remained as independent variable associated with the abdominal circumference of the newborn (*p* = 0.0270) (Table 4).

## Discussion

In this study, we have found significant associations between adipokines and insulin levels with maternal and newborn outcomes. Leptin levels increased in obese pregnant women, showing a positive correlation with maternal BMI, hyperglycemia and hypertension and an inverse correlation with newborn abdominal circumference. Adiponectin levels, in turn, were negatively correlated with gestational BMI and newborns head circumference, and positively correlated with weight at birth. Resistin levels were not associated with any maternal or fetal outcomes. Also, insulin levels were positively related to weight status and maternal hyperglycemia, but not associated with newborn outcomes. Finally, the multiple regression analysis showed that, in this pregnant women population, only maternal insulin levels were independently associated to prepregnancy BMI and maternal weight gain, with a tendency in gestational BMI (*p* = 0.0570). Likewise, only leptin levels remained as an independent variable with the abdominal circumference of the newborns.

The positive association between insulin and prepregnancy BMI (r = +0.38), gestational BMI (r = +0.24), and hyperglycemic disorders (r = +0.26) met the metabolic syndrome diagnosis criteria, whose physiopathologic basis is the association between obesity and insulin resistance [31]. The association between metabolic syndrome and gestational hyperglycemia was identified by Bo et al. [32] and recently reproduced by our research group [33, 34]. In our study the results were not different; pregnant women with overweight or obesity showed more hypertension and hyperglycemic disorders, and insulin levels were positively correlated with prepregnancy BMI, gestational BMI, and hyperglycemia. Moreover, insulin levels remained as an independent variable associated with maternal BMI and weight gain during pregnancy.

Leptin is known as the “satiety hormone” given that it inhibits food intake and stimulates energy expenditure through activation of receptors in the hypothalamus. High leptin levels are found in obese people, possibly because they exhibit resistance to the action of this hormone [17, 18]. In the present study, leptin levels were significantly higher in obese mothers, exhibiting a direct association with their BMI (r = +0.56 prepregnancy; r = +0.38 gestational period), hyperglycemic disorders (r = +0.31), and maternal hypertension (r = +0.27). These results confirm the role of leptin in energy metabolism, inflammatory state, and insulin resistance associated with obesity, that has already been found in other studies [6, 35, 36].

Adiponectin and resistin are adipocytokines with antagonistic actions. Adiponectin increases insulin sensitivity, while resistin increases insulin resistance [19, 20]. For some authors, resistin is a marker of maternal hyperglycemia, increases in late pregnancy and during the immediate post-birth period, and is associated with the HOMA-IR index [10, 15, 37, 38]. Conversely, other authors have found no differences in serum resistin levels in pregnant women with GDM, although they show higher BMI and higher levels of insulin resistance markers [39].

In the present study, the negative association between adiponectin levels and gestational BMI (r = −0.28) suggests that the higher the degree of obesity, the higher the insulin resistance, with lower adiponectin and higher resistin levels expected. Despite this, our results showed that adiponectin and resistin levels exhibited no differences among maternal BMI classes. This apparent controversy has already been described in the literature. Adiponectin concentrations were lower in insulin-resistant states such as obesity and type 2 DM [40] and pregnancy complicated by GDM [8, 23]. In contrast, adipocytokines levels (including adiponectin, leptin, and resistin), although associated with HOMA-IR, had no difference among normal or intermediate tolerance glucose and GDM pregnant women [19]. Except for leptin levels, a recent study also showed no difference in maternal levels of adiponectin and resistin between obese and non-obese pregnant women [41]. In our study, overweight or obese mothers showed 85.7 % (42/49) of hyperglycemic disorders; thus maternal obesity associated with hyperglycemia and insulin resistance could explain these results. The controversy in the literature therefore persists and may be more explored. Perhaps, the individual analysis of overweight or obese pregnant women, with and without diabetes, may solve this question.

Regarding our perinatal results, adiponectin was positively associated with fetal weight (r = +0.23) and negatively with head circumference (r = −0.27), and leptin was negatively correlated with abdominal circumference (r = −0.25). Adiponectin levels were statistically similar in normal, overweight, and obese mothers and only maternal leptin levels were statistically different and defined as an independent variable to fetal abdominal circumference. Pregnant women who are obese or have GDM typically have low circulating levels of adiponectin associated with increased fetal growth [7, 8, 42]. Therefore, the positive association between maternal adiponectin and fetal weight, as well as the negative association between maternal adiponectin and head circumference, observed in our study, was not expected.

According to a recent review, the underlying mechanisms remain largely unknown [43]. Some of these mechanisms include the action of adiponectin possibly produced by the placenta [44], the role of fetal adiponectin [45], and an antagonistic action of this hormone in the placenta inducing insulin resistance [43]. All these factors could influence and modulate fetal growth and thus alter the expected results of its biomarkers.

Corroborating our controversial findings, no association was found between maternal levels of resistin or adiponectin and weight at birth [46]. Some investigators suggested that these adipokines are markers of maternal diabetes but not of fetal weight [47], and according to others, maternal adiponectin levels are inversely related to weight at birth in both healthy pregnant women and GDM [7, 8, 13]. The maternal milieu compromised by overweight or obesity may program fat neonatal accretion, and intrauterine insulin resistance and weight gain. In this context, the evaluation of adipokines in maternal and cord blood could clarify these questions. This is one of our study limitations.

Other limitations in the present study must be considered. The sample size, probably small, should be mentioned. Maternal characteristics, with 66.7 % (48/72) presenting hyperglycemic disorders, 45.8 % (33/72) with hypertension, and 68.0 % (49/72) with overweight or obesity may have influenced the results. Targeted interventions for maternal hyperglycemia and overweight or obesity control may have also affected our results. The rates of SGA and LGA newborns in overweight (22.2 %) and obese (9.7 %) mothers, respectively; the small weight gain of overweight or obese mothers, and fetal growth markers, similar among groups, indirectly reinforce the positive effects of these interventions in our studied population.

Finally, our results have shown an independent association between maternal insulin levels and BMI status. Likewise, an independent relationship between maternal leptin and the fetal abdominal circumference was defined. To the best our knowledge, these relationships have not been presented in the literature yet, pointing new perspectives for future research. These results highlight the need of individualized evaluation considering overweight or obese mothers with and without hyperglycemic or insulin resistance conditions, not observed in most studies. Besides this, they indirectly reinforce the positive effects of specific interventions to obese and hyperglycemic mothers.

## Conclusions

Our findings support the relationship between markers of obesity and maternal–fetal outcomes. Maternal insulin and adipokines levels showed an independent relationship with respectively mother and newborns outcomes. In this studied population, the results indirectly reinforce the importance of maternal weight control before and during pregnancy to avoid adverse outcomes to mother and their newborns.
